# Sexuality and Autistic-Like Symptoms in Juvenile Sex Offenders: A Follow-Up After 8 Years

**DOI:** 10.1007/s10803-016-2805-6

**Published:** 2016-05-18

**Authors:** M. Ewoud Baarsma, Cyril Boonmann, Lisette A. ’t Hart-Kerkhoffs, Hanneke de Graaf, Theo A. H. Doreleijers, Robert R. J. M. Vermeiren, Lucres M. C. Jansen

**Affiliations:** Department of Child and Adolescent Psychiatry and the EMGO Institute for Health and Care Research, VU University Medical Center Amsterdam, Amsterdam, The Netherlands; Research and Documentation Center, Ministry of Security and Justice, The Hague, The Netherlands; Department of Forensic Child and Adolescent Psychiatry, University of Basel Psychiatric Hospital, Basel, Switzerland; Rutgers, Utrecht, The Netherlands; Institute for Criminal Justice, Faculty of Law, Leiden University, Leiden, The Netherlands; Curium-LUMC, Leiden University Medical Center, Leiden, The Netherlands

**Keywords:** Sexual offending juveniles, Autism spectrum disorder, Sexual development, Longitudinal research

## Abstract

Juveniles who have committed a sexual offense (JSOs) are thought to have abnormal sexual development, as well as increased ASD symptoms. In the current study, sexual development and behavior, as well as stability of ASD-like symptoms were assessed in a sample of 44 male JSOs (mean age 24.7 ± 1.5 years) 8 years after their sexual offence. JSOs exhibited less knowledge of sexuality, less positive attitudes towards pornography and more often reported having been a victim of verbal sexual intimidation than a matched normal population sample. ASD symptoms were relatively stable over the 8 years follow-up, indicating that social difficulties in JSOs may be part of life-long autistic-like traits. However, ASD symptoms were not related to alterations in sexual development or behavior.

**Electronic supplementary material:**

The online version of this article (doi:10.1007/s10803-016-2805-6) contains supplementary material, which is available to authorized users.

## Introduction

Sexual offending is an umbrella term, covering an array of criminal behaviors, ranging from hands-on offenses such as rape and sexual assault, to hands-off offenses such as public indecency and possession of child pornography. It is, as such, a societal problem, not only in terms of the trauma it causes its victims, but also in terms of the costs society bears, for example for prosecuting and punishing offenders (Dunsieth Jr. et al. 2004). It is suggested that a significant subgroup of sexual offenders starts their deviant sexual behavior in adolescence (Longo and Groth 1983), and approximately 20 % of all rapes and 20–50 % of child abuse cases are perpetrated by minors (Barbaree and Marshall 2008). Fortunately, not all juveniles who have committed a sexual offense (JSOs) will continue to do so in adulthood and several studies have found sexual re-offending by JSOs to be relatively rare (Caldwell 2010; Fortune and Lambie 2006). A previous study in the same group of participants by ’t Hart-Kerkhoffs et al. (2015), for example, found a sexual recidivism rate of 7 %, compared to a rate of 80 % for non-sexual recidivism. One possible explanation for this low sexual recidivism rate is that JSOs as a group may not differ from the general population with respect to psychosexual development. Previous studies have assessed some sexual domains in JSOs, such as the number of partners or deviant sexual urges (e.g., Driemeyer et al. 2013), but the sexual development of JSOs has not yet been comprehensively studied.

Besides psychosexual problems, a possible contributing factor to sexually offensive behavior in these minors is the presence of social impairments, as is frequently seen in individuals with an autism spectrum disorder (ASD). A previous study by ’t Hart-Kerkhoffs et al. (2009b) screened JSOs for the symptoms of an ASD using a parent-report questionnaire and found that JSOs display significantly more symptoms of ASDs than healthy controls. This is, however, to the best of our knowledge, the only cohort study that has assessed ASD symptoms in JSOs. Since it was cross-sectional in design, it is currently unknown whether observed ASD symptoms persist into adulthood. If autistic-like traits in adolescent JSOs are indeed based on an autism spectrum *disorder*, we would expect to see these traits persist into adulthood, since ASDs are currently understood to be life-long conditions (Matson and Horovitz 2010). Furthermore, previous research has also demonstrated that some individuals with an ASD may experience difficulties in their sexual behavior and development (Dewinter et al. 2013; Stokes et al. 2007). In JSOs, however, the relationship between ASD symptoms and sexual behavior is not yet clear. Therefore, the current study investigates the development of sexuality and ASD symptoms in JSOs from adolescence to early adulthood, as well as the relationship between these characteristics.

The foundation for a healthy sexuality in adulthood lies in childhood and adolescence, with the discovery of one’s own sexuality oftentimes going in phases (De Graaf et al. 2009). A large population study in the Netherlands amongst 7841 boys and girls aged 12–25 has shown that half of all 15-year-old adolescents have been intimate with a partner (e.g., touching each other’s genitalia). At age 16, half of all youths have experience with mutual masturbation and at age 17, half of all youths have experienced sexual intercourse and/or oral sex (De Graaf et al. 2015). Eventually, relationships and sexual intimacy become more serious; relationships last longer and are more exclusive, eventually leading to the ‘adult’ model of a committed relationship (Furman and Shaffer 2003).

Unfortunately, not all sexual behaviors that adolescents engage in are consensual. In their population study, De Graaf et al. (2012) found that 3.6 % of boys and 0.8 % of girls reported that they had ever forced another person to engage in sex. While not all of these transgressive behaviors necessarily constitute a criminal offense, some do, and a population study that looked at the criminal records of all men born in the Netherlands in the year 1984, found that 0.4 % of participants had ever been in court for a sexual offense committed during their adolescence (Lussier and Blokland 2014). While for some JSOs offending may be situational, there are several studies that suggest that for others, these offensive behaviors may be part of an atypical, or even deviant, sexual development. Galli et al. (1999) and Murphy et al. (2001), for instance, showed that some JSOs might have paraphilic desires or even pathological sexual urges, such as pathological voyeurism or pathological exhibitionism. A more recent study by Driemeyer et al. (2013), comparing JSOs and violent non-sexual offenders shortly after their arrest, also showed that JSOs exhibited sexually deviant behavior or fantasies more often. In line with other studies (Daleiden et al. 1998; Fagan and Wexler 1988), JSOs also reported to be less sexually experienced than the violent non-sexual offenders. However, these studies were mainly based on case reports or comparisons with other offenders and it has never been conclusively shown that JSOs have a different sexual development than general population youths.

Of particular interest with respect to JSOs are social difficulties that may or may not be indicative of an ASD. Disorders that fall within the autistic spectrum are characterized by both patterns of obsessive interests and stereotypical or repetitive behaviors, and problems that fall within the realm of social interaction (American Psychiatric Association 2013). Individuals with an ASD show difficulties in interacting with peers, understanding social cues, or adjusting to new situations or social settings. This frequently leads to difficulties in maintaining relationships, either with friends or with romantic and sexual partners (Stokes et al. 2007). Previous research on the sexuality of persons with an ASD has yielded various results. While several studies (Dewinter et al. 2015; Hellemans et al. 2007; Konstantareas and Lunsky 1997; Ousley and Mesibov 1991; Ruble and Dalrymple 1993; Van Bourgondien et al. 1997; Van Son-Schoones and Van Bilsen 1995) showed that persons with an ASD have wide interests in sexuality and engage in various types of normal sexual behaviors, similar to people without an ASD, others have shown that these behaviors do occur at lower rates than among typically developing peers. Mehzabin and Stokes (2011) found, for instance, that autistic individuals have less varied sexual experiences compared to typically developing peers, while Dewinter et al. (2013) in a recent review of 26 empirical studies and 29 case reports found masturbation to occur in 40–70 % of individuals with an ASD, compared to 93 % in their typically developing counterparts. Finally, a study by Marriage et al. (2009) estimated that one-third of autistic individuals had no interest in sexual activities, as compared to an estimated 1–10 % in the general population (Bogaert 2004; Poston and Baumle 2010). Normal sexual behaviors were also reported by Dewinter et al. (2013) in their review, even though atypical behaviors such as public masturbation or an attraction to pre-pubescent children were found in a small percentage of autistic individuals. One study amongst 20 individuals with an ASD (Hellemans et al. 2010) found an attraction to children in 2 individuals (10 %); another amongst 24 individuals with an ASD (Hellemans et al. 2007) reported public masturbation in 3 individuals (13 %). Other studies (Ginevra et al. 2016; Stokes and Kaur 2005, Stokes et al. 2007) have also found individuals with an ASD to display significantly more inappropriate sexual and romantic behaviours than normal controls. Examples of such atypical behaviors included focusing one’s affection on celebrities or strangers, or pursuing love interests for an inappropriately long period of time. In the study by Stokes et al. (2007), parents of individuals with an ASD reported that when they confronted their children with the inappropriateness of their courting behaviors, their children “did not believe they were doing anything wrong” and “couldn’t understand why the person wouldn’t respond to them as they wanted” (Stokes et al. 2007, p. 1978). Stokes et al. (2007) also found a relationship between social functioning and the success of an intimate relationship. Persons with an ASD were found to rely less on peers and friends than non-ASD individuals with regard to social and romantic learning (Stokes et al. 2007).

While the dyadic behaviors referenced above (Stokes et al. 2007) are oftentimes harmless, sometimes they may become so transgressive that they constitute a sexual offense (Scragg and Shah 1994; Howlin 2004; Stokes et al. 2007). Howlin (2004) suggests that specific symptoms of autistic individuals, such as obsessively pursuing certain interests, not understanding social cues and rigid adherence to one’s own routine, may put them at greater risk of committing a crime than people from the general population. Symptoms of an ASD have further been associated with violent outbreaks by ASD individuals (Scragg and Shah 1994; Howlin 2004). This supports the hypothesis of Realmuto and Ruble (1999) that the social difficulties of persons with an ASD could lead to a misinterpretation of social situations, which in turn could lead to transgressive behaviors or even sexual delinquency. To the best of our knowledge, however, there are currently no quantitative studies showing increased sexually violent behavior in ASD patients. A study on JSOs on the other hand has shown that there is a significantly higher degree of ASD-like social problems in JSOs than in juveniles from the general population, especially in JSOs harassing children and in soloist peer offenders (’t Hart-Kerkhoffs et al. 2009b). In sum, we would conclude that, based on our review of the literature, a possible contributing factor in JSOs committing sexual offenses may be the presence of ASD-like social difficulties, as observed by ’t Hart-Kerkhoffs et al. (2009b).

It is currently unknown, however, if these social problems in JSOs are merely of a temporary nature and related to the situation and life phase of the adolescent at the time of the offense, or if they are stable over time and may even be indicative of an ASD. Since previous studies were cross-sectional (e.g., ’t Hart-Kerkhoffs et al. 2009b), the question whether the ASD symptoms of JSOs persist into adulthood remains unanswered. Taking into account previous research, these ASD symptoms may also influence sexual development and behaviors of JSOs, and also possibly disturbances therein. Therefore, stability of the ASD symptoms will be related to the assessment of sexuality and sexual development at follow-up. Moreover, since little is known about whether the sexuality and sexual development of JSOs deviates from that of the normal population, a comparison of sexuality and sexual development of JSOs and the normal population is needed.

Therefore, the aim of the current study is to gain a better insight into the sexual development, as well as the longitudinal development of symptoms of an ASD in JSOs. Specific objectives were to assess (1) whether the sexuality and sexual development of young adults who have committed a sexual offense in adolescence differs from that of normal controls, (2) whether ASD-like symptoms in JSOs persist into adulthood, and (3) whether sexuality and sexual development of JSOs are related to (the persistence of) ASD-like symptoms. Based on previous studies (e.g., Driemeyer et al. 2013), we hypothesized that JSOs would differ from normal controls with respect to sexual development; for instance, that they would engage in sexual behavior at a later age and at lower rates than normal controls. Furthermore, we hypothesized that this difference would be more outspoken in the JSOs that had persistently high levels of ASD symptoms.

## Methods

### Participants and Procedure

Participants in this prospective longitudinal study were 44 males who, during their adolescence, had been arrested in the Netherlands between May 2003 and December 2006 (T0) as suspects of a sexual offense and at that time had been assessed by researchers from our department. All juveniles (now young adults) were revisited approximately 8 years later between September 2013 and August 2014 (T1) and examined again.

The sample at T0 (’t Hart-Kerkhoffs et al. 2009b) consisted of 175 boys (mean age 14.49 ± 1.4 years). As police officers in the Netherlands are required to refer all under-aged suspects of a sexual offense to the Child Protection Board (CPB; Raad voor de Kinderbescherming), participants were primarily included through the CPB. In addition, sex offenders on remand at pre-trial juvenile detention centers were also included. Both the CPB offices and the juvenile detention centers had been selected for their location spread over urban and rural regions. Although all participants were only suspects at the time of inclusion, all participants will be referred to as JSOs for reasons of readability. Exclusion criteria were an IQ below 70 or insufficient proficiency in the Dutch language. Due to the limited number of female JSOs, all females were excluded.

At the time of inclusion at T0, all participants and their parents or legal representatives were given detailed information about the study, after which their written informed consent was acquired. All participants were then given the T0 questionnaires to fill out.

**Fig. 1:**
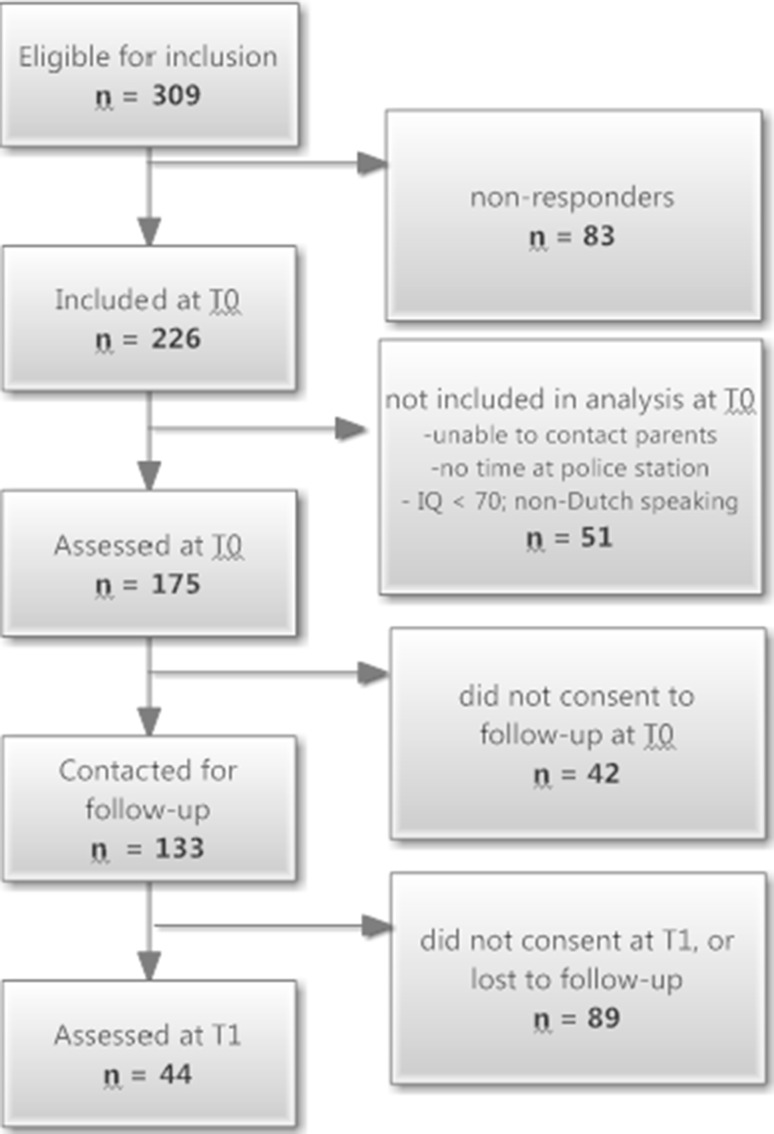
Enrollment and follow-up

Of the 175 boys included at T0, 133 (76 %) had at that time given their informed consent to be contacted in the future for follow-up studies. At the time of the follow-up (T1), the researchers tried to contact these 133 boys using letters and house visits, asking them if they were willing to participate in the present follow-up study. Of the 133 boys, 44 (response rate = 33 %) agreed to participate at T1, while the remaining JSOs declined to participate (*n* = 25; 19 %) or were lost to follow-up because their whereabouts could not be traced (*n* = 64; 48 %) (Fig. 1).

The mean follow-up time for all participants assessed at T1 was 8.6 years (*SD* = 0.8 years). All participants at T1 were informed in detail about the follow-up study through an explanatory letter and a phone call or house visit, after which written informed consent for the follow-up study was acquired. Participants were then given login codes to a secure Internet environment, which held the questionnaires. This course of action was preferable to letting participants come to the institution, so as to make the study less burdensome for them. Furthermore, Turner et al. (1997) showed that youths are more inclined to share sensitive personal information in a computerized questionnaire, than in an interview or in a questionnaire on paper. Participants at T1 were paid around €30,00 as compensation for the use of their time.

Participants and non-participants at T1 did not differ with respect to current age [*t*(180) = 0.228; *p* = 0.820], ethnicity [χ^2^(1) = 3.104, *p* = 0.100], type of inclusion site [χ^2^(1) = 0.075; *p* = 0.758], offense characteristics, such as gender of victim [χ^2^(2) = 0.896; *p* = 0.639], age of victim [χ^2^(1) = 1.243; *p* = 0.293], type of index offense (child abuse, soloist peer offense or group sex offense) [χ^2^(2) = 0.024; *p* = 0.363], non-sexual recidivism rate [χ^2^(1) = 1.501; *p* = 0.249], sexual recidivism rate [χ^2^(1) = 0.068; *p* = 1.000], or total days spent in detention [participants: *n* = 30, *M* = 463, *SD* = 687; non-participants: *n* = 125, *M* = 459, *SD* = 608; *t*(153) = −0.033, *p* = 0.973]. The data on detention were current to September 12th, 2011 and included any day spent incarcerated, whether it be related to the index offense, or to any other criminal charge.

The study was approved by the Medical Ethics Committee of the VU University Medical Center, Amsterdam, The Netherlands.

### Control Group

The sexual development and sexuality variables of JSOs measured at T1 were compared to those of a control group consisting of 52 young males, drawn from a larger representative sample of 7841 adolescents and young adults. This larger sample was studied by De Graaf et al. (2012) as part of a population study on the sexual health of Dutch youth. Participants were recruited either through schools, or by sending them a written invitation using addresses acquired through governmental records. Both schools and individual addresses were randomly selected so as to yield an even spread of participants across rural and urban areas (De Graaf et al. 2012). The control group was matched with participants on a group level with respect to age, ethnic background and level of education (Table 1). Since the control group was drawn from the database of the aforementioned study by De Graaf et al. (2012), which focused only on sexuality and sexual development, ASD symptoms and prior criminal history in the control group could not be assessed.

**Table 1:** Population characteristics

	JSOs (n = 44)	Controls (n = 52)	t/χ^2^	*p* value
Age at T1 (in years)	24.7 (SD = 1.5)	24.3 (SD = 0.7)	1.760	0.084
Education level^a^				
Lower	16 (40 %)	21 (40 %)	0.297	0.862
Middle	20 (50 %)	24 (46 %)		
Higher	4 (10 %)	7 (14 %)		
Ethnic background^b^				
Dutch	24 (56 %)	31 (60 %)	0.140	0.835
Non-Dutch	19 (44 %)	21 (40 %)		

^a^For 4 JSOs, education level was unknown

^b^For 1 JSO, ethnic background was unknown

### Instruments

#### Assessment of Sexuality

Sexuality and sexual development were assessed *retrospectively* during follow-up in early adulthood, using the SOJ25II (Seks onder je 25^e^-II [Sex under the age of 25-II] (De Graaf et al. 2012)) questionnaire.

The SOJ25II is a questionnaire from Rutgers WPF (the Netherlands’ leading expertise center on sexuality), based on multiple validated questionnaires about sexuality and sexual development. These questionnaires have been translated into Dutch and adapted to fit the language proficiency of vocational students. The SOJ25II is very valuable, since it currently is the most complete questionnaire on the sexuality and sexual development of adolescents and young adults in the Dutch language. It assesses amongst others the age at which juveniles reach certain sexual milestones, communication about sexuality with peers and adults, sexual victimization and coercion, knowledge of sex and attitudes towards sexuality. Moreover, the SOJ25II was used by Rutgers in a representative sample (*N* = 7841) of Dutch adolescents up to the age of 25. Mean scale scores were calculated for positive attitudes towards sexuality (Cronbach’s α = 0.79), body image (α = 0.59) and sexual self-image (α = 0.76); positive attitudes towards pornography (α = 0.61), as well as discussing sexuality with parents (α = 0.91) and friends (α = 0.89). The specific questions that made up each scale can be found in the Supplementary Appendix.

#### Assessment of Autism Spectrum Symptoms

ASD symptoms were assessed *longitudinally* at adolescence (at the time of the sexual offence, T0) as well as during follow-up in early adulthood (T1).

At T0, symptoms of an ASD were assessed by means of the Child Social Behaviour Questionnaire (*in Dutch: VISK, Vragenlijst voor de Inventarisatie van Sociaal gedrag bij Kinderen*) (Hartman et al. 2006, 2007a, b). The CSBQ is filled out by parents or caregivers and includes 49 items with a 3-point Likert-scale divided on 6 subscales (tuned behavior, social behavior, orientation in time and place, understanding, stereotyped behavior, reaction to change) covering a broad range of features typically seen in children with an ASD. The CSBQ has been shown to have good psychometric properties with regard to reliability (internal consistency: α = 0.94; inter-rater reliability: ICC = 0.86; test–retest reliability *r* = 0.90) (Hartman et al. 2006), and moderate validity (Pearson *r* ranging from 0.30 to 0.63), with respect to both high-functioning autists and autistic individuals with a mild to moderate intellectual disability (De Bildt et al. 2009; Hartman et al. 2006; Luteijn et al. 2000).

At follow-up (T1), symptoms of an ASD were assessed by means of the Adult Social Behaviour Questionnaire (*in Dutch: VIS*-*V, Vragenlijst voor de Inventarisatie van Sociaal gedrag bij Volwassenen*) (Hartman et al. in press). It consists of both a self-report questionnaire for the participant to fill out and a questionnaire to be completed about the participant by a third person (e.g., a partner, parent, or other relative). The ASBQ includes 44 items with a 3-point Likert-scale divided in 6 subscales (reduced contact, reduced empathy, reduced interpersonal insight, violation of social conventions, insistence on sameness and sensory stimulation/motor stereotypies) and covers a broad range of features typically seen in adults with an ASD. The ASBQ can be regarded as the adult version of the CSBQ.

### Data and Statistical Analysis

The data were analyzed using IBM SPSS 19 (International Business Machines Corporation Statistical Package for the Social Sciences, version 19). Prior to analysis, the data were screened for missing values, normality and outliers. Missing values and outliers were excluded on a per-analysis basis.

For all calculations, the level of statistical significance was set at 0.05. First, using Chi squares and independent *t* tests, the sexual experiences, and the attitudes towards and communication about sexual issues of JSOs were compared to those of normal controls. Where values were not normally distributed, non-parametric tests were used. Second, the development of symptoms of an ASD over time was analyzed using bivariate correlation. Because scores were not normally distributed, Spearman’s rank correlation coefficients were calculated. Finally, the relationship between the symptoms of an ASD and the several measurements around sexuality was analyzed. In order to analyze the correlation between the continuous CSBQ/ASBQ-scores and the continuous variables of the SOJ25II-questionnaire bivariate correlations were used; for the correlation between CSBQ/ASBQ-scores and dichotomous variables of the SOJ25II-questionnaire *t* tests were used.

## Results

### Milestones in the Sexual Development of JSOs as Compared to Normal Controls

No differences were found between JSOs and normal controls with regard to the number of individuals in each group that had ever engaged in a certain type of sexual behavior (Table 2). JSOs were found to have started with anal sex at a younger age than normal controls [JSO: *M* = 17.4 years, *SD* = 2.6; NC: M = 20.5 years, *SD* = 2.3; *t*(24) = − 3.199, *p* = 0.004]. For all other forms of sexual behavior, no significant differences were found. Finally, there were no differences between JSOs and normal controls in the existence or duration of a current relationship or in the number of sex partners they had had throughout their lives.

**Table 2:** Milestones in the sexual development of JSOs compared to normal controls

	JSOs (n = 44)		Controls (n = 52)		χ^2^ (yes/no)	t (start age)
	n (%)	Mean start age (SD)	n (%)	Mean start age (SD)		
Being in a relationship	33 (88)	16.2 (3.4)	44 (85)	15.8 (3.1)	0.281 *p* = 0.766	0.504 *p* = 0.616
Kissing with tongue	42 (98)	13.6 (2.4)	47 (90)	14.6 (3.6)	2.114 *p* = 0.217	−1.517 *p* = 0.133
Feeling and petting	41 (95)	13.9 (2.3)	47 (90)	14.4 (3.4)	0.850 *p* = 0.451	−0.841 *p* = 0.403
Manual sex (passive)	38 (88)	14.8 (2.5)	43 (87)	15.3 (3.0)	0.604 *p* = 0.565	−0.951 *p* = 0.345
Manual sex (active)	39 (90)	14.7 (2.0)	45 (87)	15.8 (3.2)	0.398 *p* = 0.749	−1.934 *p* = 0.050
Vaginal sex	38 (80)	15.5 (2.4)	45 (87)	16.4 (2.5)	0.072 *p* = 1.000	−1.746 *p* = 0.085
Oral sex^a^ (passive)	41 (98)	15.8 (2.5)	45 (94)	16.4 (2.7)	0.790 *p* = 0.620	−1.059 *p* = 0.293
Oral sex^a^ (active)	35 (83)	16.6 (2.9)	41 (85)	16.7 (2.4)	0.074 *p* = 1.000	−0.049 *p* = 0.961
Anal sex^a^	13 (31)	17.4 (2.6)	13 (27)	20.5 (2.3)	0.163 *p* = 0.816	−3.199 *p* = 0.004

	JSOs (n = 44)	Controls (n = 52)	χ^2^/t
Currently in a relationship	23 (54)	26 (50)	0.115 *p* = 0.837
Duration of current relationship	<3 months: 2 (9) <1 year: 1 (4) ≥1 year: 20 (87)	<3 months: 1 (4) <1 year: 3 (12) ≥1 year: 22 (85)	1.250 *p* = 0.535
Mean no. of sex partners^b^	11.9 (SD = 15.5)	6.9 (SD = 9.4)	1.855 *p* = 0.068

^a^For JSOs, n = 42; for controls, n = 48

^b^For JSOs, n = 42

### Knowledge, Attitudes and Communication with Respect to Sexuality, JSOs Compared to Normal Controls

Testing knowledge of sex and sexuality, JSOs scored significantly lower (*M* = 3.86, *SD* = 2.50) than normal controls (*M* = 5.33, *SD* = 2.31), *t*(93) = −2.67, *p* = 0.004 (Table 3). JSOs and controls were found not to differ with respect to the attitudes they had towards sex, nor with respect to their sexual self-image or body image. JSOs were found to hold less positive opinions about pornography than controls [JSO: *M* = 3.14, *SD* = 0.62; NC: *M* = 3.44, *SD* = 0.64; *t*(85) = −2.139, *p* = 0.032]. When confronted with questions about sex, JSOs were not found to consult any sources (e.g., parents, peers, official sources) more or less often than normal controls did. When asked whom they went to when they had a problem regarding sexual issues, JSOs reported significantly less often that they could talk to their partner, χ^2^(1) = 5.370, *p* = 0.031. This relationship remained statistically significant when only the JSOs or controls who were currently engaged in a relationship were included in the analysis [JSO: 6 of 23 (26 %) said ‘yes’; normal controls: 20 of 26 (77 %) said ‘yes’; χ^2^(1) = 2.663, *p* = 0.001]. Analyses of other related variables in this category yielded no significant results.

**Table 3:** Knowledge, attitudes and communication with respect to sexuality, JSOs compared to normal controls

	JSOs (n = 43)	Controls (n = 52)	t	*p* value
Knowledge regarding sexuality^a^	3.86 (SD = 2.50)	5.33 (SD = 2.31)	−2.967	0.004
Positive attitudes towards sex^b,c^	4.24 (SD = 0.50)	4.30 (SD = 0.51)	−0.591	0.555
Positive body image^b,c^	3.34 (SD = 0.72)	3.28 (SD = 0.61)	0.470	0.644
Positive self-image^b,c^	4.13 (SD = 0.49)	4.24 (SD = 0.47)	−1.014	0.315
Positive attitudes towards pornography^b^	3.14 (SD = 0.62)	3.43 (SD = 0.64)	−2.139	0.035
Discussing sexuality with parents before the age of 16^d^	1.71 (SD = 0.69)	1.69 (SD = 0.84)	0.149	0.882
Discussing sexuality with friends^d^	1.98 (SD = 0.75)	2.03 (SD = 0.95)	−0.251	0.802

	*n* (%) who said ‘yes’	*n* (%) who said ‘yes’	χ^2^	*p* value
When I have questions about sex, I consult:				
My parents	9 (21)	10 (20)	0.042	1.000
Siblings	4 (9)	5 (10)	0.003	1.000
Friends	15 (35)	25 (48)	1.681	0.216
Professionals (e.g. doctor, youth worker)	15 (35)	18 (35)	0.001	1.000
Other sources (e.g. internet)	32 (74)	44 (85)	1.576	0.303
No-one	6 (14)	3 (6)	1.838	0.291
I never have questions about sex	0 (0)	3 (6)	2.562	0.249
When I have a problem regarding sex, I can talk to:				
My partner	10 (23)	24 (46)	5.370	0.031
My parents	11 (26)	11 (21)	0.259	0.633
Siblings	5 (12)	7 (14)	0.072	1.000
Friends	11 (26)	20 (29)	1.776	0.196
Professionals (e.g. doctor, youth worker)	8 (19)	12 (23)	0.283	0.624
Others	5 (12)	7 (14)	0.072	1.000
No-one	5 (12)	6 (12)	0.000	1.000
I never have problems regarding sex	13 (30)	11 (21)	1.027	0.349
I can not be part of my group of friends if I:^e^				
Am not in a relationship	1 (3)	1 (2)	0.117	0.943
Have never kissed with tongue	2 (6)	1 (2)	1.213	0.545
Have never had sex	2 (6)	1 (2)	0.761	0.684
Don’t have sex with a lot of different people	2 (6)	1 (2)	0.761	0.684
I think it’s wrong if/to:^f^				
A girls hits on a guy	4 (9)	2 (4)	1.184	0.405
A girl has sex with a lot of guys	24 (56)	32 (62)	0.319	0.676
A guy hits on a girl	1 (2)	1 (2)	0.019	1.000
A guy has sex with a lot of girls	20 (47)	28 (54)	0.507	0.539
Give someone drugs or alcohol in order to get sex	42 (98)	51 (98)	0.019	1.000
Force someone to have sex	42 (98)	52 (100)	1.222	0.453

	JSOs (n = 41)	Controls (n = 46)		
How many good friends do you have?				
None	4 (10)	1 (2)	3.418	0.332
1	7 (17)	11 (24)		
2	10 (24)	8 (17)		
3 or more	20 (49)	26 (57)		

^a^Mean score on 7 knowledge questions. A correct answer was scored +1 point, an incorrect answer was scored −1 point and ‘don’t know’ was scored 0 points. For the questions, see the addendum

^b^Mean score on a Likert-scale: ‘strongly disagree (1) through ‘strongly agree’ (5). Questions were positively and negatively formulated. Negatively formulated questions were recoded. A high score indicates a positive attitude. For the questions, see the addendum

^c^For JSOs, n = 41; for normal controls n = 48

^d^Mean score on a Likert-scale: ‘never’ (1) through ‘very often’ (5). For the separate items, see the addendum

^e^Responses ‘strongly agree’ and ‘agree’ were counted as ‘yes’; ‘don’t know’ was counted as a missing value

^f^Responses ‘strongly agree’ and ‘agree’ were counted as ‘yes’

### Sexual Victimization of and Sexual Coercion by JSOs as Compared to Normal Controls

JSOs and normal controls reported that they perceived themselves as victims of sexual coercion in 7% and 4 % of cases respectively (Table 4). When asked more specifically, 43 % of JSOs and 25 % of controls reported being the victim of an event that constituted sexual intimidation, sexual assault, or rape. When split out for the several types of sexual victimization, these rates were 27 and 14 % respectively for sexual assault, and 9 and 2 % respectively for rape. JSOs reported having been a victim of verbal sexual intimidation significantly more often than normal controls [χ^2^(1) = 4.494, *p* = 0.039]; for physical sexual intimidation there were no significant differences. When comparing various variables comparing JSOs and normal controls as perpetrators of sexual coercion and intimidation, no significant differences were found.

**Table 4:** Sexual victimization of, and sexual coercion by JSOs compared to normal controls

	JSOs (n = 44)	Controls (n = 52)	χ^2^	*p* value
	n (%)	n (%)		
Reported perceiving themselves as a victim of sexual coercion in general	3 (7)	2 (4)	0.426	0.658
Reported having been the victim of an event that constitutes^a,b^				
Verbal sexual intimidation	16 (36)	9 (17)	4.494	0.039
Physical sexual intimidation	6 (14)	4 (8)	0.902	0.505
Sexual assault	12 (27)	7 (14)	2.864	0.124
Rape	4 (9)	1 (2)	2.480	0.176
Any form of victimization (combined)	19 (43)	13 (25)	3.545	0.082
Reported perceiving themselves as a perpetrator of sexual coercion in general	6 (14)	4 (8)	0.902	0.505
Reported having committed an act that constitutes^b^				
Verbal sexual intimidation	14 (32)	17 (33)	0.008	1.000
Physical sexual intimidation	2 (5)	2 (4)	0.029	1.000

^a^In this table, sexual assault is defined as involuntary kissing, intimate touching or manual sex; rape is defined as involuntary oral, vaginal or anal sex

^b^In this table, verbal sexual intimidation is defined as any form of non-violent psychological pressure onto the victim with the intent to make him/her consent to sex; physical sexual intimidation is defined as the use of, or threat of violence in order to have sex, as well as taking advantage a situation where the victim is under the influence of alcohol or drugs

### Symptoms of an ASD in JSOs at T0 and T1

Mean ASD symptom scores on the CSBQ (T0) and the ASBQ (T1) are given in Table 5. Statistically significant correlations were found between the CSBQ and the ASBQ (other-report) (ρ = 0.453, *p* = 0.006), and the ASBQ (self-report) and ASBQ (other-report) (ρ = 0.684, *p* < 0.001). The correlation between the CSBQ and the ASBQ (self-report) was not statistically significant.

**Table 5:** Mean per-item CSBQ and ASBQ-scores and correlations

	n	Mean per-item score	SD	CSBQ (other)	ASBQ (self)	ASBQ (other)
T0						
CSBQ (other)	43	0.507	0.377	1		
T1						
ASBQ (self)	44	0.442	0.316	0.295 *p* = 0.061	1	
ASBQ (other)	39	0.407	0.350	0.453 *p* = 0.006	0.684 *p* = <0.001	1

All items could be scored 0 (not present), 1 (a bit present), or 2 (clearly present)

### Correlation of Symptoms of an ASD and Sexuality

When comparing the scores on the CSBQ and ASBQ of JSOs that had engaged in certain sexual behaviors to those that had not, a significant relationship was found for feeling and petting [*CSBQ* yes: *M* = 0.462, *SD* = 0.350; no: *M* = 1.214, *SD* = 0.188; *t*(34) = 1.582, *p* = 0.023] and manual active sex (*CSBQ* yes: *M* = 0.453, *SD* = 0.349; no: *M* = 1.075, *SD* = 0.276; *t*(38) = 2.994, *p* = 0.011), albeit with a ‘no’-group of *n* = 2 and *n* = 3 respectively (Supplementary Table S1a). For both types of behavior, the ‘no’-group scored higher on the CSBQ than the ‘yes’-group. CSBQ and ASBQ scores did not differ with respect to other types of sexual behavior. Correlating the mean age at which JSOs had started engaging in certain sexual behaviors to their CSBQ and ASBQ scores also yielded no significant results (Supplementary Table S1b). The same was true for the correlation between the amount of sex partners and CSBQ/ASBQ scores. CSBQ scores of the JSOs that are currently in a relationship were significantly lower than those that were not in a relationship [yes: *M* = 0.376, *SD* = 0.257; no: *M* = 0.667, *SD* = 0.456; *t*(23.46) = − 2.368, *p* = 0.014].

Assessing the communication of JSOs correlated to the symptoms of an ASD, several significant relationships were found (Supplementary Table S2). The CSBQ scores of those that did not talk to their friends when they had a question about sex were significantly higher than those that did [yes: *M* = 0.322, *SD* = 0.263; no: *M* = 0.596, *SD* = 0.402; *t*(38) = 2.293, *p* = 0.027]; their ASBQ-scores were also higher, but not significant. No significant differences were found in the CSBQ/ASBQ-scores with respect to the persons JSOs talk to when they have problems related to sexuality, or the topics they discuss with their friends or parents, nor were there any significant differences in CSBQ/ASBQ-scores with respect to the sexual norms in their group of friends.

Assessing the knowledge of JSOs of sexuality, no correlation was found between their knowledge of sexuality and their CSBQ/ASBQ-scores (Supplementary Table S3). Assessing societal norms around sexuality, JSOs that reported objecting to same-sex public displays of affection between girls had significantly lower CSBQ-scores than those that did not object [yes: *M* = 0.314, *SD* = 0.188; no: *M* = 0.546, *SD* = 0.402; *t*(24.88) = 2.378, *p* = 0.025]. All other analyses with respect to this topic yielded no significant results, nor were any significant differences found with respect to the correlation between positive attitudes towards sexuality, body image or pornography and CSBQ/ASBQ-scores.

As for sexual victimization of JSOs and by JSOs (Supplementary Table S4), those that reported perceiving themselves a victim of sexual coercion, had significantly higher scores on both the ASBQ (self) [yes: *M* = 0.886, *SD* = 0.444; no: *M* = 0.409, *SD* = 0.286; *t*(42) = − 2.703, *p* = 0.010] and ASBQ (other) [yes: *M* = 0.917, *SD* = 0.568; no: *M* = 0.365, *SD* = 0.300; *t*(37) = −2.868, *p* = 0.007], albeit with *n* = 3 (supplementary Table 10). When asked specifically about whether they had been a victim of an event that constituted either sexual intimidation, or sexual assault, or rape, JSOs that answered in the affirmative (*n* = 19) had significantly higher scores on the ASBQ (other) [yes: *M* = 0.550, *SD* = 0.375; no: *M* = 0.284, *SD* = 0.279; *t*(37) = − 2.534, *p* = 0.016]. When splitting out the several forms of sexual victimization, those that reported an event that constituted sexual assault (*n* = 12) and rape (*n* = 4), also had significantly higher ASBQ (self) [*for sexual assault:* yes: *M* = 0.655, *SD* = 0.364; no: *M* = 0.362, *SD* = 0.259; *t*(15.38) = − 2.561, *p* = 0.021 *and for rape:* yes: *M* = 0.909, *SD* = 0.365; no: *M* = 0.395, *SD* = 0.274; *t*(42) = − 3.480, *p* = 0.001] and ASBQ (other)-scores [*for sexual assault*: yes: *M* = 0.640, *SD* = 0.432; no: *M* = 0.315, *SD* = 0.259; *t*(13.14) = − 2.320, *p* = 0.037 *and for rape* yes: *M* = 0.829, *SD* = 0.499; no: *M* = 0.359, *SD* = 0.302; *t*(37) = − 2.723, *p* = 0.010]. The same result was found for those that reported having been a victim of physical sexual intimidation [*ASBQ* (*self*): yes: *M* = 0.799, *SD* = 0.349; no: *M* = 0.385, *SD* = 0.275; *t*(42) = − 3.309, *p* = 0.002 *and ASBQ* (*other*): yes: *M* = 0.767, *SD* = 0.767; no: *M* = 0.341, *SD* = 0.341; *t*(37) = − 3.027, *p* = 0.004], but not for verbal sexual intimidation. No significant differences in CSBQ/ASBQ-scores were found with respect to those that reported having been the perpetrator of sexual coercive or intimidating behavior.

## Discussion

The aim of this study was to gain a better insight into the longitudinal development of symptoms of an ASD in JSOs, as well as into their sexual development. We found that there was a strong positive correlation between the ASD-like symptoms of JSOs at T0 and T1. This is an indication that observed social difficulties are not a temporary phenomenon, but are a stable trait and may, for some JSOs, be indicative of the life-long presence of autistic traits, or even an autism spectrum disorder. With respect to the various domains of sexuality we studied, however, JSOs and controls were actually found to be quite similar. Also, our study showed no relations between sexual behaviors and ASD-like symptoms.

Our findings that JSOs and normal controls are quite similar in terms of sexuality, for example, with respect to achieving sexual milestones, communicating about and attitudes towards sex, seem to contrast previous studies (Fagan and Wexler 1988; Daleiden et al. 1998; Driemeyer et al. 2013), which found that JSOs were, for instance, less sexually active than controls. However, in those studies, the period of time between the offense and the study was relatively short: participants were studied shortly after their arrest, during the adjudication process or while incarcerated. The discrepancy between our results and those of previous studies could therefore be partly explained by the extended period of time between the initial study and our follow-up. Either the maturation of the JSOs, or interventions during that follow-up time might explain why JSOs and controls are currently much alike with respect to many aspects of sexuality. Another possible explanation lies in that fact that Daleiden et al. (1998) and Fagan and Wexler (1988) studied JSOs who were incarcerated, as opposed to our sample of JSOs, of whom most had not been incarcerated for quite some time at the time of the study. A lack of opportunity to engage in sexual behavior on the part of the participants in studies referenced above may explain the discrepancy between our results and theirs. Finally, Driemeyer et al. (2013) and Fagan and Wexler (1988) did not compare the JSOs to normal controls, but to violent non-sexual offenders. It is, therefore, possible that JSOs are not necessarily *less* sexually experienced, but violent non-sexual offenders could in actuality be *more* sexually experienced. This hypothesis is supported by the fact that the ages at which JSOs reached certain sexual milestones did not differ from the ages of sexual milestones found in the Dutch general population (De Graaf et al. 2015).

With respect to attitudes towards sexual issues, we observed no major differences between JSOs and controls, most notably with respect to peer communication, nor did we find a correlation between the degree of ASD-like symptoms and whether JSOs talked to peers about sexuality. This result seems to conflict with the study results by Stokes et al. (2007), who found that persons with an ASD relied less on peer communication as a source for sex-related information. It has to be noted that Stokes et al. (2007) studied individuals with an actual diagnosis of ASD, while we only screened for ASD symptoms; this might explain the indicated differences. We also found that JSOs scored lower than controls on sexual knowledge. These findings seem to be in line with the findings of ’t Hart-Kerkhoffs et al. (2009a), who found that at T0, the majority of JSOs in our group had had insufficient sex education. While a lack of knowledge on the prevention of sexually transmitted infections and pregnancy may of course not be related to sexually offensive behavior, it may be indicative, however, of a general lack of sexual education and formation, including education on engaging in normal sexual relations.

A sexual domain where we did find significant differences between JSOs and controls was sexual victimization. ’t Hart-Kerkhoffs et al. (2009a) already found at T0 that 12 % of the JSOs studied were sexually victimized themselves and at T1, this rate has only gone up. JSOs more often than controls reported being victimized, however, only for verbal sexual intimidation this difference was significant. We also found that only very few respondents reported that they had committed an act of sexual coercion themselves. This finding is remarkable considering the fact that out of the 226 JSOs whose offenses at T0 were indexed for this research project, 156 youth were convicted by the courts (conviction rate = 69 %). Our findings at T1 could therefore be explained by recall bias, the persistent belief that one’s own actions had not constituted an offense, intentionally dishonest answers, or the fact that normal controls exhibit sexually transgressive behavior just as often as JSOs, but are less often apprehended by police. With respect to the victimization of JSOs, the chance of becoming a victim of sexual coercion seemed to be related to the degree of ASD symptoms JSOs displayed: for both sexual assault and rape, as well as physical sexual intimidation, ASBQ-scores in the victimized group were on average twice as high as in the group that was not victimized. These findings seem to corroborate the recent study by Brown-Lavoie et al. (2014), who found that individuals with an ASD were two to three times more likely to become victims of sexual coercion than individuals without an ASD. Considering the fact that JSOs in general are already at risk of being victimized, this renders the JSO with an ASD especially vulnerable to sexual violence.

With respect to the stability of ASD symptoms in JSOs, we found that there was a strong positive correlation between the number of ASD symptoms that JSOs display at the time of the index offense (T0) and the number of ASD symptoms they display at follow-up (T1). This result, however, was found only in the *other*-reports; the correlation between the *self*-*report* part of the ASBQ and the CSBQ was not statistically significant. Our findings suggest that the ASD-related problems present at the time of the offense were not temporary, but that these persist into later life. Since we have not conducted a diagnostic assessment, we may not conclude that these findings prove an increased prevalence of ASDs in JSOs. However, these findings may be an indication that it is important to further study autism spectrum disorders in juveniles who are suspected of having committed a sexual offense.

Finally, we found no relation between sexuality and sexual development on the one hand, and ASD symptoms on the other. However, it should be noted that the literature on sexuality and ASDs in non-delinquent youth consists of both quantitative (e.g., Dewinter et al. 2015) and qualitative studies (e.g., Dewinter et al. 2016). To the best of our knowledge, however, sexuality and ASDs amongst JSOs have to date only been studied with a quantitative design, as we have done in the current study. As a result, we may have missed subtle changes in sexual cognition and behavior that may be related to ASD symptoms. Future studies should include qualitative methods as well to get more insight in the relation between social difficulties and sexuality and sexual development.

### Limitations

Our results have to be viewed in light of some limitations. First, all data were collected through questionnaires. Even though combining self-report and other-report questionnaires renders our results more valid, these screening questionnaires are not a substitute for a diagnostic tool. It was, therefore, impossible to make clinical diagnoses of an ASD. This means we cannot make conclusions concerning the prevalence of ASDs in JSOs. Second, data on sexuality were collected retrospectively at T1 and not at the time of the offense. This means that with respect to several variables, we could not assess whether events related to that variable occurred before, during or after the index offense. Hence, we could not compare whether JSOs and controls differed in terms of their sexual behavior at the time of the offense, but only whether they were different at follow-up, 8 years later. This is relevant, since JSOs will have had some form of treatment, guidance or counseling after their contact with the criminal justice system, which may have had a positive influence on their sexual development. Since we do not have precise information about the treatment they received, we cannot correct for this. Third, a large portion of the sample at T0 was for various reasons lost to follow-up, mostly because their whereabouts could not be traced. This might render the follow-up sample less comparable to the initial sample. Both groups, however, did not differ with respect to many relevant factors, such as ethnicity or offense characteristics. Also, a problem inherent in most forensic research is that it always difficult to assess whether a sample is representative of all offenders: some JSOs included may actually be innocent and vice versa, a whole subset of offenders may have been missed because they were never apprehended by police. Finally, considering the relatively small sample size, certain subtle differences may have been missed, including those between the several subgroups of JSOs (child molesters, solo peer offenders and group offenders). Previous studies (e.g., ’t Hart-Kerkhoffs 2009b) have found significant differences in ASD symptoms between these subgroups.

### Clinical and Scientific Implications

To the best of our knowledge, this is the first long-term longitudinal study assessing the psychosexual development of juveniles who sexually offend with a follow-up period of this length. While many questions surrounding the mental health and sexual development of JSOs remain unanswered, especially on the prevalence of ASDs in JSOs, this study has, in our view, made some important points. With respect to sexual development and behaviors, our study has shown that approximately 8 years after the index offense, there are few differences between JSOs and normal controls. We cannot, however, make any conclusions as to whether there were significant differences at the time of the offense, and if so, what has caused these differences to disappear. Nonetheless, the fact that JSOs have committed a sexual offense, apparently does not preclude them from having a normal sexual development in the long term. This is important to know both for the juvenile, whose burden of being labeled a ‘sexual predator’ can be diminished, as well as for professionals in the justice system and mental health care system. Whether through normal development or psychosocial interventions, for most JSOs, things do at least seem to work out alright with respect to the areas of sexual development we studied. Furthermore, our finding that ASD symptoms in JSOs persist over time again underlines the necessity to be alert of autistic traits in JSO. Signaling these autistic traits can lead prosecutors and the courts to initiate treatment to improve social difficulties, and to offer education and guidance in the area of sexuality. That way, we may even further reduce the (already low) recidivism amongst JSOs. Finally, our findings with respect to sexual victimization further underline the fact that JSOs, and especially JSOs with more pronounced ASD symptoms, are not only perpetrators of sexual offenses, but are also frequently the victims thereof.

We would recommend further research on JSOs to look more closely into ASD-specific sexual problems, or subtle psychosexual disturbances. The SOJ25II questionnaire is a general questionnaire testing normal sexual development with little attention to these specific issues. Further research may benefit from a qualitative setup to ascertain whether these subtle problems exist. Finally, there is still no conclusive answer to the question if there is a higher prevalence of ASDs among JSOs as compared to the general population. Future studies should use a comprehensive diagnostic tool to assess this prevalence.

## Electronic supplementary material

Below is the link to the electronic supplementary material.

Supplementary material 1 (DOCX 36 kb)
