# Supplementary material for: Sexuality and Autistic-Like Symptoms in Juvenile Sex Offenders: A Follow-Up After 8 Years
Source: J Autism Dev Disord. 2016 May 18;46:2679–91. doi: 10.1007/s10803-016-2805-6 (PMC4938848; doi:10.1007/s10803-016-2805-6)
Supplement: Supplementary file 1 — Supplementary material 1 (DOCX 36 kb) [file 10803_2016_2805_MOESM1_ESM.docx]

**ADDENDA**

**Table 3: Seven knowledge questions about sexuality**

1. A girl can become pregnant if the boy doesn’t orgasm during [vaginal] sex.
2. The contraceptive pill reduces the chance of contracting HIV or another STI.
3. If you wash yourself well after sex, you will contract HIV or another STI less easily.
4. If you don’t have any physical symptoms, you can still have an STI.
5. There are STI’s which cause girls to no longer be able to have children when they’re older.
6. If you take the contraceptive pill, you will no longer be able to have children when you’re older.
7. A girl always bleeds the first time she has [vaginal] sex.

**Table 3: Eleven statements regarding body image and attitudes towards sex, listed by scale**

*Attitudes towards sex (negative statements were recoded)*

1. I’m embarrassed if I (were to) have sexual feelings.
2. I think sex is gross.
3. I feel guilty if I (were to) have sexual feelings.
4. I feel guilty after masturbating.
5. Sex is important to me.
6. I want to try all sorts of things with respect to sex
7. I think sex is nice.

*Body image (negative statements were recoded)*

1. I think I’m pretty attractive
2. I’m content with my genitalia
3. I think I’m too fat or too thin.
4. I’d like to be more muscular.

Total combined self-image was a combination of both scales.

**Table 3: Eight statements regarding positive attitudes towards pornography**

**(negative statements were recoded)**

1. Most porn is gross.
2. Porn can be arousing.
3. In porn, you see what women like
4. In porn, women are often treated not very nicely.
5. In porn, you see what men like.
6. You can learn a lot from porn.
7. Watching porn is bad.
8. Sex in porn isn’t the same as real sex.

**Table 3: Discussing sexuality with parents: six topics**

1. Love and relationships
2. Sexual things you like to do
3. Sexual things you do not like to do
4. How to prevent pregnancy
5. How to prevent STI’s
6. How to prevent negative sexual experiences
7. Homosexuality

**Table 3: Discussing sexuality with friends: five topics**

1. Love and relationships
2. Sexual things you like to do
3. Sexual things you do not like to do
4. How to prevent pregnancy
5. How to prevent STI’s
6. How to prevent negative sexual experiences

**SUPPLEMENTARY TABLES**

| **TABLE S1a: Milestones in sexual development of JSOs, correlation with ASD symptoms: experience** | | | | | | | | | | | | | |
| --- | --- | --- | --- | --- | --- | --- | --- | --- | --- | --- | --- | --- | --- |
|  | Mean score on CSBQ  (SD) | | | t | | Mean score on ASBQ (self) (SD) | | t | Mean score on ASBQ (other) (SD) | | t | | |
| Experience with… | ‘yes’ | | ‘no’ |  |  | ‘yes’ | ‘no’ |  | ‘yes’ | ‘no’ |  |  |  |
| Being in a relationship | 0,5000  (0,38355) | | 0,4980  (0,39325) | -0,011  p=0,991 | | 0,4408  (0,32057) | 0,4636  (0,34862) | 0,148  p=0,883 | 0,4242 (0,35847) | 0,2557  (023864) | -0,912  p=0,368 | | |
| Kissing with tongue | 0,4848  (0,37251) | | 1,0816  (n/a) | 1,582  p=0,122 | | 0,5909  (0,32266) | 0,4399  (n/a) | 0,462  p=0,646 | n/a | 0,4069  (0,34950) | n/a | | |
| Feeling and petting | 0,4621  (0,34914) | | 1,2143  (0,18760) | 2,998  p=0,005 | | 0,4313  (0,32167) | 0,6932  (0,14464) | 1,136  p=0,263 | 0,3933  (0,34352) | 0,9250  (n/a) | n/a | | |
| Manual sex (passive) | 0,4611  (0,35090) | | 0,8469  (0,50830) | 2,001  p=0,053 | | 0,4408 (0,32673) | 0,4636  (0,29087) | 0,148  p=0,883 | 0,3969 (0,35361) | 0,4949  (0,34439) | 0,526  p=0,602 | | |
| Manual sex (active) | 0,4531  (0.34944) | | 1,0748  (0,27557) | 2,994  p=0,005 | | 0,4347 (0,32462) | 0,6144  0,30367) | 0,554  p=0,583 | 0,3896  (0,35122) | 0,6144  (0,30367) | 1,072  p=0,291 | | |
| Vaginal sex | 0,4755  (0,57241) | | 0,6694  (0,34917) | 1,070  p=0,291 | | 0,4474  (0,32842) | 0,4136  (0,27149) | -0,219  p=0,827 | 0,4067  (0,35444) | 0,4091  (0,35139) | 0,013  p=0,990 | | |
| Oral sex ^a^  (passive) | 0,4621  (0,34914) | | 1,3469  (n/a) | 2,502  p=0,017 | | 0,4313 (0,32167) | 0,7955  (n/a/ | 1,119  p=0,270 | 0,3933  (0,34352) | 0,9250  (n/a) | 1,528  p=0,135 | | |
| Oral sex ^a^  (active) | 0,4908 (0,35480) | | 0,4524  (0,49746) | 0,229  p=0,820 | | 0,4416  (0,33362) | 0,4318  (0,28386) | -0,072  p=0,943 | 0,4063  (0,34240) | 0,4097  (0,40973) | 0,023  p=0,982 | | |
| Anal sex ^a^ | 0,4798 (0,28420) | | 0,4867 (0,40666) | 0,051  p=0,959 | | 0,3916 (0,33236) | 0,4616  (0,32176) | 0,645  p=0,522 | 0,3624 (0,25087) | 0,4244 (0,38402) | 0,494  p=0,624 | | |
|  | Mean score on CSBQ | | | t / ρ | |  | | t / ρ |  | | t / ρ | | |
|  | ‘yes’ | | ‘no’ |  | |  |  |  |  |  |  | | |
| Currently in a relationship | 0,3759  (0,25735) | | 0,6672  (0,45646) | -2,368  p=0,014 | | 0,4012  (0,31645) | 0,4920  (0,32438) | -0,928  p=0,359 | 0,3810  (0,37911) | 0,4405  (0,31519) | -0,522  p=0,605 | | |
| Mean no. of sex partners^b^ |  | | | -0,075  p=0,634 | |  | | -0,056  p=0,720 |  | | -0,153  p=0,352 | | |
|  | |  | | |  | | | | | | |  |  |

| **TABLE S1b: Milestones in sexual development of JSOs, correlation with ASD symptoms: starting age** | | | | | | | | | | |
| --- | --- | --- | --- | --- | --- | --- | --- | --- | --- | --- |
|  |  | | CSBQ | | | | ASBQ (self) | | ASBQ (other) | |
|  | mean start age  (SD) | | Spearman’s ρ | p-value | | | Spearman’s ρ | p-value | Spearman’s ρ | p-value |
| Being in a relationship | 16,2 (3,4) | | -0,031 | 0,847 | | | -0,011 | 0,943 | -0,247 | 0,130 |
| Kissing with tongue | 13,6 (2,4) | | 0,008 | 0,962 | | | -0,067 | 0,673 | -0,111 | 0,500 |
| Feeling and petting | 13,9 (2,3) | | -0,034 | 0,840 | | | 0,039 | 0,809 | -0,025 | -0,879 |
| Manual sex (passive) | 14,8 (2,5) | | -0,142 | 0,409 | | | 0,041 | 0,807 | 0,056 | 0,749 |
| Manual sex (active) | 14,7 (2,0) | | -0,169 | 0,317 | | | 0,041 | 0,807 | 0,046 | 0,788 |
| Vaginal sex | 15,5 (2,4) | | -0,115 | 0,512 | | | 0,016 | 0,923 | 0,052 | 0,767 |
| Oral sex ^a^  (passive) | 15,8 (2,5) | | -0,026 | 0,875 | | | -0,121 | 0,452 | -0,138 | 0,409 |
| Oral sex ^a^  (active) | 16,6 (2,9) | | 0,027 | 0,879 | | | 0,044 | 0,802 | 0,069 | 0,706 |
| Anal sex ^a^ | 17,4 (2,6) | | -0,178 | 0,600 | | | -0,503 | 0,080 | -0,079 | 0,819 |
|  | |  | | |  |  |  |  |  |  |

| **TABLE S2: Communication about sexuality of JSOs, correlation with ASD symptoms** | | | | | | | | | | | |
| --- | --- | --- | --- | --- | --- | --- | --- | --- | --- | --- | --- |
|  | Mean score on CSBQ  (SD) | | t / ρ | Mean score on ASBQ (self) (SD) | | t / ρ | Mean score on ASBQ (other) (SD) | | | | t / ρ |
|  | ‘yes’ | ‘no’ |  | ‘yes’ | ‘no’ |  | ‘yes’ | ‘no’ | | |  |
| When I have questions about sex, I consult:  my parents  siblings  friends  officials (e.g. doctor, youth worker)  other sources (e.g. internet)  no-one  I never have questions about sex | 0,6386  (0,44375)  0,4847  (0,49273)  0,3216  (0,26264)  0,4198  (0,39311)  0,5108  (0,37484)  0,3216  (0,14884)  n/a  (n/a) | 0,4594  (0,35689)  0,5014  (0,37383)  0,5956  (0,40202)  0,5427  (0,37278)  0,4616  (0,40406)  0,5251  (0,39670)  0,4997  (0,37962) | -1,256  p = 0,217  0,082  p = 0,935  2,293  p = 0,027  0,976  p = 0,335  -0,338  p = 0,737  2,154  p = 0,049  n/a  n/a | 0,4672  (0,40942)  0,3125  (0,25738)  0,3318  (0,26765)  0,3394  (0,25125)  0,4148  (0,30305)  0,5909  (0,44883)  n/a  (n/a) | 0,4372  (0,29865)  0,4569  (0,32510)  0,5032  (0,33337)  0,4992  (0,34197)  0,5269  (0,36616)  0,4195  (0,29495)  0,4434  (0,21962) | -0,248  p = 0,806  0,858  p = 0,396  1,715  p = 0,094  1,591  p = 0,119  1,003  p = 0,322  -1,226  p = 0,227  n/a  n/a | 0,5261  (0,50837)  0,2841  (0,32699)  0,2727  (0,26013)  0,3279  (0,31302)  0,4420  (0,35501)  0,3450  (0,23219)  n/a  (n/a) | 0,3762  (0,29942)  0,4210  (0,35371)  0,4821  (0,37465)  0,4563  (0,36817)  0,3178  (0,33424)  0,4160  (0,26532)  0,4069  (0,34950) | | | -0,799  p=0,446  0,738  p = 0,465  1,851  p = 0,072  1,121  p = 0,270  -0,999  p = 0,324  0,420  p = 0,667  n/a  n/a |
| When I have a problem regarding sex, I can talk to:  my partner  my parents  siblings  friends  officials (e.g. doctor, youth worker)  others  no-one  I never have problems regarding sex | 0,5533  (0,40360)  0,5299  (0,37500)  0,6964  (0,50788)  0,5653  (0,43580)  0,4235  (0,36438)  0,3827  (0,71134)  0,3396  (0,16553)  0,4494  (0,38096) | 0,4842  (0,37789)  0,4882  (0,38731)  0,4716  (0,35841)  0,4778  (0,36454)  0,5188  (0,38659)  0,5127  (0,33981)  0,5175  (0,39367)  0,5213  (0,38397) | -0,476  p = 0,637  -0,306  p = 0,761  -1,248  p = 0,220  -0,626  p = 0,535  0,630  p = 0,532  0,361  p = 0,741  0,887  p = 0,381  0,544  p = 0,590 | 0,3841  (0,25879)  0,5000  (0,38596)  0,3727  (0,26968)  0,4483  (0,38917)  0,3551  (0,16296)  0,4455  (0,36597)  0,7045  (0,39365)  0,3671  (0,27160) | 0,4614  (0,33736)  0,4240  (0,29804)  0,4528  (0,32762)  0,4418  (0,29923)  0,4636  (0,34420)  0,4432  (0,31857)  0,4091  (0,29797)  0,4765  (0,33718) | 0,666  p = 0,509  -0,676  p = 0,503  0,522  p = 0,605  0,317  p = 0,954  1,325  p = 0,198  -0,015  p = 0,988  -2,012  p = 0,171  0,479  p = 0,308 | 0,4180  (0,29010)  0,4645  (0,45779)  0,3932  (0,43974)  0,5250  (0,41108)  0,2782  (0,23995)  0,5023  (0,43919)  0,3450  (0,23219)  0,3950  (0,35914) | 0,4031  (0,37238)  0,3871  (0,31116)  0,4085  (0,34559)  0,3606  (0,31846)  0,4351  (0,36611)  0,3929  (0,34015)  0,4160  (0,36532)  0,4116  (0,35223) | | | -0,114  p = 0,910  -0,599  p = 0,553  0,082  p = 0,935  -1,336  p = 0,190  1,078  p = 0,288  -0,648  p = 0,521  0,420  p = 0,677  0,131  p = 0,896 |
| Discussing sexuality with parents before the age of 16 ^a^ |  | | 0,232  p = 0,161 |  | | -0,083  p = 0,606 |  | | | | 0,092  p = 0,581 |
| Discussing sexuality with friends ^a^ |  | | -0,228  p = 0,195 |  | | -0,273  p = 0,101 |  | | | | -0,330  p = 0,057 |
| I cannot be a part of my group of friends if I ^b^:  am not in a relationship  have never kissed with tongue  have never had sex  don’t have sex with a lot of different  people | 0,1633  (n/a)  0,1429  (0,02886)  0,1429  (0,02886)  0,1429  (0,02886) | 0,4673  (0,36616)  0,4788  (0,36669)  0,4788  (0,36669)  0,4788  (0,36669) | 0,817  p = 0,420  1,276  p = 0,212  1,276  p = 0,212  1,276  p = 0,212 | 0,2045  (n/a)  0,2955  (0,12856)  0,2955  (0,12856)  0,2955  (0,12856) | 0,4024  (0,28865)  0,4029  (0,29311)  0,4029  (0,29311)  0,4029  (0,29311) | 0,676  p = 0,504  0,510  p = 0,614  0,510  p = 0,614  0,510  p = 0,614 | 0,1364  (n/a)  0,2273  (0,12856)  0,2273  (0,12856)  0,2273  (0,12856) | 0,3841  (0,20602)  0,3863  (0,31100)  0,3863  (0,31100)  0,3863  (0,31100) | | | 0,797  p = 0,432  0,710  p = 0,483  0,710  p = 0,483  0,710  p = 0,483 |
| a: Mean score on a Likert-scale: ‘never’ (1) through ‘very often’ (5). For the separate items, see the addendum.  b: Responses ‘strongly agree’ and ‘agree’ were counted as ‘yes’; ‘don’t know’ was counted as a missing value. | | | | | | | | |  |  |  |

| **TABLE S3: Knowledge of and attitudes towards sexuality of JSOs, correlation with ASD symptoms** | | | | | | | | | | | |
| --- | --- | --- | --- | --- | --- | --- | --- | --- | --- | --- | --- |
|  | CSBQ | | | ASBQ (self) | | | ASBQ (other) | | | | |
|  | Spearman’s ρ | | p-value | Spearman’s ρ | | p-value | Spearman’s ρ | | | | p-value |
| Knowledge regarding sexuality ^a^ | 0,042 | | 0,797 | -0,134 | | 0,392 | -0,124 | | | | 0,452 |
| Positive attitudes towards  sex ^b, c^  Positive body image ^b, c^  Positive self-image ^b, c^ | -0,151  -0,050  -0,162 | | 0,364  0,767  0,331 | -0,185  -0,152  -0,186 | | 0,246  0,341  -0,245 | -0,260  -0,303  -0,227 | | | | 0,114  0,065  0,170 |
| Positive attitudes towards  pornography ^b^ | 0,100 | | 0,550 | 0,129 | | 0,422 | -0,006 | | | | 0,973 |
|  | Mean score on CSBQ (SD) | | t | Mean score on ASBQ (self) (SD) | | t | Mean score on ASBQ (other) (SD) | | | | t |
|  | ‘yes’ | ‘no’ |  | ‘yes’ | ‘no’ |  | ‘yes’ | | ‘no’ | |  |
| I think it’s wrong if/to ^d^  a girls hits on a guy  two girls kiss in public  a girl has sex with a lot of guys  a guy and a girl kiss in public  a guy hits on a girl  a guy has sex with a lot of girls  two guys kiss in public  give someone drugs or alcohol  in order to get sex  force someone to have sex | 0,2959  (0,19751)  0,3144 (0,18803)  0,5459  (0,39619)  0,2819  (0,12903)  0,3265  (n/a)  0,5819  (0,40484)  0,4262  (0,24184)  0,4958  (0,38376)  0,4958  (0,38376) | 0,5224  (0,38983)  0,5460 (0,40280)  0,4372  (0,35809)  0,5239  (0,39132)  0,5041  (0,38353)  0,4175  (0,34307)  0,5662  (0,46755)  0,6531  (n/a)  0,6531  (n/a) | 1,136  p = 0,263  2,378  p = 0,025  -0,893  p = 0,377  1,217  p = 0,231  0,457  p = 0,650  -1,385  p = 0,174  1,205  p = 0,237  0,405  p = 0,688  0,405  p = 0,688 | 0,5114 (0,45625)  0,4943 (0,30775)  0,4782  (0,33358)  0,5000  (0,35501)  1,0000  (n/a)  0,4841  (0,34382)  0,3898  (0,26904)  0,4518  (0,31866)  0,4518  (0,31866) | 0,4365 (0,30975)  0,4318  (0,32550)  0,3995  (0,30419)  0,4360  (0,31914)  0,4302  (0,31131)  0,4081  (0,30022)  0,4901  (0,35724)  0,0909  (n/a)  0,0909  (n/a) | -0,442  p = 0,661  -0,494  p = 0,624  -0,798  p = 0,429  -0,417  p = 0,679  -1,809  p = 0,078  -0,774  p = 0,443  1,028  p = 0,310  -1.119  p = 0,269  -1.119  p = 0,269 | 0,4631 (0,48313)  0,5420  (0,30914)  0,4936  (0,36725)  0,4727  (0,14287)  0,5000  (n/a)  0,5158  (0,32888)  0,3841  (0,30966)  0,4111  (0,35323)  0,4111  (0,35323) | | 0,4005 (0,33987)  0,3824  (0,35507)  0,3058  (0,30702)  0,4015  (0,36201)  0,4045  (0,35385)  0,3228  (0,34875)  0,4265  (0,38692)  0,2500  (n/a)  0,2500  (n/a) | | -0,335  p = 0,739  -1,030  p = 0,310  -1,715  p = 0,095  -0,335  p = 0,739  -0,266  p = 0,791  -1,756  p = 0,087  0,374  p = 0,711  -0,450  p = 0,655  -0,450  p = 0,655 |
| a: Mean score on 7 knowledge questions. A correct answer was scored +1 point, an incorrect answer was scored -1 point and ‘don’t know’ was scored 0 points. For the questions, see the addendum.  b: Mean score on a Likert-scale: ‘strongly disagree (1) through ‘strongly agree’ (5). Questions were positively and negatively formulated. Negatively formulated questions were recoded. A high score indicates a positive attitude. For the questions, see the addendum.  c: For JSOs, n=41; for normal controls n = 48  d: Responses ‘strongly agree’ and ‘agree’ were counted as ‘yes’. | | | | | | | |  | |  |  |

| **TABLE S4: Sexual victimization of and sexual coercion by JSOs, correlation with ASD symptoms** | | | | | | | | | | | |
| --- | --- | --- | --- | --- | --- | --- | --- | --- | --- | --- | --- |
|  | Mean score on CSBQ  (SD) | | t | Mean score on ASBQ (self) (SD) | | t | Mean score on ASBQ (other) (SD) | | | | t |
|  | ‘yes’ | ‘no’ |  | ‘yes’ | ‘no’ |  | ‘yes’ | ‘no’ | | |  |
| Reported perceiving themselves victim of sexual coercion in general | 0,7245  (0,10102) | 0,4978  (0,38559) | -0,821  p = 0,417 | 0,8864  (0,44362) | 0,4091  (0,28577) | -2,703  p = 0,010 | 0,9167  (0,56833) | 0,3645  (0,30006) | | | -2,868  p = 0,007 |
| Combined reported victimization of an event that constitutes either sexual intimidation, or sexual assault, or rape | 0,5522 (0,38435) | 0,3627 (0,27387) | -0,611  p = 0,545 | 0,5455 (0,34451) | 0,3627 (0,27387) | -1,961  p = 0,057 | 0,5503 (0,37564) | 0,2841 (0,27895) | | | -2,534  p = 0,016 |
| Reported being the victim of an event that constitutes ^a^  sexual assault  rape | 0,5937  (0,34495)  0,6395  (0,16369) | 0,4778  (0,39215)  0,4986  (0,39074) | -0,864  p = 0,393  -0,614  p = 0,542 | 0,6553  (0,36439)  0,9091  (0,36505) | 0,3615  (0,25922)  0,3949  (0,27436) | -2,561  p = 0,021  -3,480  p = 0,001 | 0,6399  (0,43236)  0,8239  (0,49978) | 0,3154  (0,25922)  0,3594  (0,30281) | | | -2,320  p = 0,037  -2,723  p = 0,010 |
| Reported having been a victim of ^b^  verbal sexual intimidation  physical sexual intimidation | 0,5075  (0,32497)  0,7755  (0,25975) | 0,5097  (0,41373)  0,4800  (0,38153) | 0,017  p = 0,986  -1,503  p = 0,141 | 0,4801  (0,33524)  0,7992  (0,34897) | 0,4196  (0,30875)  0,3852  (0,27507) | -0,606  p = 0,548  -3,309  p = 0,002 | 0,4967  (0,37649)  0,7674  (0,40669) | 0,3508  (0,32711)  0,3414  (0,30069) | | | -1,278  p = 0,209  -3,027  p = 0,004 |
| Reported having committed an act of sexual coercion in general | 0,3776  (0,20962) | 0,5314  (0,39915) | 1,412  p = 0,183 | 0,4167  (0,31415) | 0,4456  (0,32044) | 0,206  p = 0,841 | 0,3782  (0,15304) | 0,4112  (0,37105) | | | 0,195  p = 0,847 |
| Reported having committed  an act of ^b^  verbal sexual intimidation  physical sexual intimidation | 0,4869  (0,27728)  0,6224  (0,33191) | 0,5203  (0,42734)  0,5030  (0,38459) | 0,302  p = 0,765  -0,430  p = 0,670 | 0,3734  (0,24956)  0,2955  (0,09642) | 0,4735  (0,34198)  0,4486  (0,32169) | 0,978  p = 0,334  0,665  p = 0,510 | 0,3934  (0,26151)  0,2057  (0,09803) | 0,4137  (0,39077)  0,4178  (0,35539) | | | 0,169  p = 0,866  0,833  p = 0,410 |
| a: In this table, sexual assault is defined as involuntary kissing, intimate touching or manual sex; rape is defined as involuntary oral, vaginal or anal sex.  b: In this table, verbal sexual intimidation is defined as any form of non-violent psychological pressure onto the victim with the intent to make him/her consent to sex; physical sexual intimidation is defined as the use of or threat of violence in order to have sex, as well as taking advantage a situation where the victim is under the influence or alcohol or drugs. | | | | | | | | |  |  |  |
